# Iron uptake in quiescent and inflammation-activated astrocytes: A potentially neuroprotective control of iron burden

**DOI:** 10.1016/j.bbadis.2013.04.007

**Published:** 2013-08

**Authors:** Ilaria Pelizzoni, Daniele Zacchetti, Alessandro Campanella, Fabio Grohovaz, Franca Codazzi

**Affiliations:** a San Raffaele Scientific Institute, via Olgettina 60, 20132 Milano, Italy; b San Raffaele University, via Olgettina 58, 20132 Milano, Italy

**Keywords:** NTBI, non-transferrin-bound-iron, TRP, transient receptor potential, DMT1, divalent metal transporter 1, BBB, blood–brain barrier, Tf, transferrin, TfR1, transferrin receptor 1, TBI, Tf-bound iron, FAS, ferrous ammonium sulfate, FAC, ferric ammonium citrate, VOCCs, voltage-operated calcium channels, PLC, phospholipase C, DHPG, dihydroxyphenylglycine, IL, interleukin, TNF, tumor necrosis factor, LIP, labile iron pool, IFN, interferon, TIRF microscopy, total internal reflection fluorescence microscopy, EYFP, enhanced yellow fluorescent protein, NMDA, N-Methyl-d-aspartate, TRPV1, transient receptor potential vanilloid 1, Astrocytes, Non-transferrin-bound iron uptake, DMT1, Activation process, TRP channels, Neuroinflammation

## Abstract

Astrocytes play a crucial role in proper iron handling within the central nervous system. This competence can be fundamental, particularly during neuroinflammation, and neurodegenerative processes, where an increase in iron content can favor oxidative stress, thereby worsening disease progression. Under these pathological conditions, astrocytes undergo a process of activation that confers them either a beneficial or a detrimental role on neuronal survival. Our work investigates the mechanisms of iron entry in cultures of quiescent and activated hippocampal astrocytes. Our data confirm that the main source of iron is the non-transferrin-bound iron (NTBI) and show the involvement of two different routes for its entry: the resident transient receptor potential (TRP) channels in quiescent astrocytes and the de novo expressed divalent metal transporter 1 (DMT1) in activated astrocytes, which accounts for a potentiation of iron entry. Overall, our data suggest that at rest, but even more after activation, astrocytes have the potential to buffer the excess of iron, thereby protecting neurons from iron overload. These findings further extend our understanding of the protective role of astrocytes under the conditions of iron-mediated oxidative stress observed in several neurodegenerative conditions.

## Highlights

• Non-transferrin-bound iron (NTBI) is the main source of iron for astrocytes.
• TRPC channels represent an entry pathway for Fe^2 +^ in resting astrocytes.
• Activation process increases the competence of astrocytes to uptake iron.
• DMT1 expression accounts for potentiation of iron ingress in activated astrocytes.

## 1 Introduction

Astrocytes are versatile cells with a wide range of physiological functions. They have long been known to contribute not only to the formation of the Blood–brain Barrier (BBB) but also to provide trophic support to neurons and to regulate the synaptic microenvironment. More recently, they have been proposed to modulate neuronal activity and control neuroinflammation [1], [2]. Within this complex framework, astrocytes participate to brain homeostasis of iron, an equally versatile element, which is essential not only for a wide variety of physiological functions but which is also able to induce oxidative damages when mishandled. Indeed, astrocytes control brain iron uptake through the BBB and are responsible for iron redistribution to neuronal cells. Several reports indicate that iron exceeds the binding capacity of Transferrin (Tf) in brain interstitial fluids, thus implying that a significant amount of iron circulates free or loosely bound to carrier molecules (e.g. ATP, ascorbate, citrate) released by the astrocytes [3], [4]. This NTBI pool is considered to be the main source of iron for astrocytes in vivo, since the expression of Tf receptor 1 (TfR1) and the uptake of Tf-bound-iron (TBI) have been reported only in culture [5], [6]. NTBI uptake was suggested to occur via the DMT1, the main transporter responsible for Fe^2 +^ intake in mammals [7]; nonetheless, there is only limited evidence of its expression in vivo, at the level of the astrocytic perivascular endfeet [8], [9], while its cellular distribution and specific plasma membrane localization in cultured astrocytes have yet to be clearly demonstrated [10], [11]. Other mechanisms, such as the zinc transporter Zip14, have been proposed for NTBI uptake in astrocytes [7], [12], but even in this case their physiological role is still to be established.

More recently, reports have shown that Fe^2 +^ uptake can occur via calcium permeable channels in different cell types, a possibility that might have strong physiopathological implications not only for neurons but also for astrocytes [13], [14], [15]. The pathways responsible for NTBI uptake in astrocytes are still matter of debate: there is wide consensus that NTBI enters mainly as Fe^2 +^, while little evidence supports the existence of a Fe^3 +^ import pathway in astrocytes in vitro [16], [17].

Many studies indicate that the NTBI pool increases in pathological conditions: in acute brain injury, such as hemorrhagic stroke [18]; in several neurodegenerative disorders, causing the oxidative stress involved in disease progression [12], [19]; in autoimmune diseases, such as multiple sclerosis [20]. All these pathological conditions are associated with neuroinflammation, a complex response to the cytokines and the pro-inflammatory molecules released by microglia. As a consequence, also astrocytes undergo changes in their phenotype, in a process known as activation. Activated astrocytes surround brain lesions undergoing neurodegeneration and modulate the inflammatory response, with possible neuroprotective or detrimental effects on the neighboring neurons [21].

Although it is well established that inflammation influences systemic iron metabolism, little is known about the effects of neuroinflammation on brain iron homeostasis. In this study we characterize the mechanisms responsible for NTBI uptake in primary hippocampal astrocytes in resting conditions as well as upon inflammatory activation. Our final aim was to investigate whether the activation process could improve the capability of astrocytes to handle and buffer the NTBI pool, thereby protecting neurons by a potentially dangerous outcome [15].

## 2 Materials and methods

### 2.1 Cell cultures

Primary rat hippocampal astrocytes were prepared from 2 to 3 day-old Sprague–Dawley rats, according to [15]. The Institutional Animal Care and Use Committee of the San Raffaele Scientific Institute approved the experimental procedures.

Pure astrocyte cultures were obtained by two steps of overnight shaking at 200 rpm; selective detachment of microglia was confirmed by the absence (< 0.1%) of staining for IBA1, a specific microglia marker [22]. Confluent astrocytes were trypsinized and re-plated onto poly-lysine-coated coverslips or Petri dishes and experiments were performed within 3 days after re-plating. In order to obtain the activated phenotype, the astrocytes were treated with cytokines [23]. A mix of recombinant rat interleukin-1β (IL1β; 10 ng/ml) and tumor necrosis factor α (TNFα; 30 ng/ml) or interferon γ (INFγ; 20 ng/ml) was administered to astrocytes and incubated for 24 h at 37 °C. The cytokines were from R&D Systems (Minneapolis, MN, USA).

### 2.2 Videomicroscopy

The videomicroscopy setup is based on an Axioskope 2 microscope (Zeiss, GmbH, Martinsried, Germany) and a Polychrome IV (Till Photonics, GmbH, Martinsried, Germany) light source. The total internal reflection fluorescence (TIRF) microscopy setup was described in [24]. The ratio analysis was performed between the fluorescence signals (evaluated within the same region of interest) from TIRF and epifluorescence.

Fura-2 acetoxymethyl ester (Calbiochem, Merck KGaA, Darmstadt, Germany) and calcein acetoxymethyl ester (Molecular Probes, Life Technologies, Carlsbad, CA, USA) loadings were performed at 37 °C (4 μM 40 min and 0.25 μM 3 min, respectively) in Krebs Ringer Hepes buffer (KRH, containing 5 mM KCl, 125 mM NaCl, 2 mM CaCl_2_, 1.2 mM MgSO_4_, 1.2 mM KH_2_PO_4_ and 6 mM glucose, 20 mM Hepes, pH 7.4). Single cell experiments were performed in KRH buffer at room temperature. To monitor Fe^2 +^ variations, fura-2 was excited at 355 nm. This wavelength was adopted as isosbestic since it turned out to be Ca^2 +^ insensitive in our optical configuration [15].

### 2.3 Pharmacological treatments

Fe^2 +^ and Fe^3 +^ water stock solutions were freshly prepared by dissolving ferrous ammonium sulfate and ferric ammonium citrate (Sigma-Aldrich, St. Louis, MO, USA), respectively. In some experiments cells were pre-treated for the specified times with pharmacological agents (Sigma-Aldrich) listed as follows: ebselen (50 μM, a DMT1 blocker), for 40 min; nimodipine (10 μM, a dihydropyridine l-type VOCC blocker), verapamil (100 μM, a phenylalkylamine l-type VOCC blocker), SC38249 (100 μM, a TRPC blocker) and LU52396 (10 μM, a TRPC blocker), for 15 min; oATP (100 μM, a blocker of P2X7 receptors) for 1 h.

### 2.4 Real time PCR analysis

RNA was extracted from cells with TRIzol (Invitrogen, Life Technologies) following manufacturer instruction. Single strand cDNA was obtained using Superscript III Retrotranscription Kit (Invitrogen) with random hexamers as primers. SYBR green-based reverse transcription quantitative PCR (RT-qPCR) was performed and analyzed on a LightCycler 480 (Roche Diagnostics, Basel, Switzerland). Specific primers were: GTCCGATGGGGAAGAAGCA forward for DMT1-1A, CCTGGGATATGGGGTCGC forward for DMT1-1B, GTGAAGGGCTCCTCAGAATC reverse for both DMT1 1A and B; GCCTGTCTGTCTGTCTTTGC and CCCAGTGTTTCCCAACTAACA for DMT1-IRE(+), TAGATGACCAACAGCCCAGA and CACAGCCGTTAGCTTTACCC for DMT1-IRE(−); TCACCATTAAGCTGGGCG and TTCTTCCCGGTCCAGTCATA for frataxin (used for normalization).

### 2.5 LIP measurement

Cells were loaded with calcein and the fluorescence was measured before and after 15 min incubation with 100 μM salicylaldehyde isonicotinoyl hydrazone (SIH), a cell permeant iron chelator. The analysis was performed by using a High Throughput Microscopy (HTM) system, the IN Cell Analyzer 1000 [15] (GE Healthcare, Grandview Blvd, Waukesha, WI, USA).

### 2.6 ^55^Fe uptake

To evaluate iron uptake, astrocytes (sampled in triplicate) were incubated 18 h with 2 μM ^55^Fe–Ammonium Citrate (Perkin Elmer, Monza, Italy), corresponding to 2.5 μCi/ml, in the presence of 1 μM ascorbic acid (Sigma-Aldrich). For the higher Fe^2 +^ concentration, 2 μM ^55^Fe–Ammonium Citrate was mixed with 18 μM of non-radiolabelled Fe–Ammonium Citrate. Cells were then washed three times with phosphate-buffered saline and lysed with 20 mM Tris–HCl pH 7.4 with 0.5% Triton X-100. Cellular extracts were collected and centrifuged at 16,000 *g* for 10 min. Samples (10 μl) from the soluble fraction were mixed with 0.5 ml of Ultima Gold (Packard Instrument Co, Meriden, CT) and counted (3 min) in a scintillation counter (Packard Instrument Co). Finally, total protein content of soluble cellular extracts was used to normalize radioactive counts.

### 2.7 Expression vectors and cell transfection

The pEYFP-C1-DMT1-1A/IRE(+) and pEYFP-C1-DMT1-1B/IRE(+) vectors were generated as described in [25]. Primary hippocampal astrocytes were transfected using Lipofectamine 2000 (Invitrogen) according to the manufacturer's instructions. Cells were analyzed 24–48 h after transfection.

### 2.8 Western blotting

Cells were lysed by mechanical scraping in ice-cold PBS containing 0.1 mM EDTA, 2% Nonidet P-40, 0.2% sodium dodecyl sulfate (SDS) and CLAP. Samples (20 μg of proteins per lane) in denaturating buffer (50 mM Tris/HCl, 2.5 mM EDTA/Na, 2% SDS, 5% glycerol, 20 mM DTT, 0.01% bromophenol blue) were incubated 10 min at 65 °C and proteins separated by standard SDS—polyacrylamide gel electrophoresis (SDS-PAGE) and electrically transferred onto nitrocellulose membrane. Membranes were blocked with Tris–Buffered Saline (TBS) supplemented with 0.1% Tween-20 and 5% skimmed milk powder. Primary antibodies were diluted as follows: mouse anti-TfR1 antibody, 1:3000 and rabbit anti-actin 1:5000 (Invitrogen) in blocking solution; rabbit anti-DMT1 antibody 1:500 in TBS—0.1% Tween-20. After washing, membranes were incubated with secondary goat anti-rabbit or anti-mouse HRP-conjugated antibodies (Biorad, Hercules, CA, USA) diluted 1:2000 in blocking solution. Protein bands were detected on autoradiographic films by chemiluminescence with the West Pico or West Femto Super Signal substrate (Pierce, Thermo Fisher Scientific, Waltham, MA, USA).

### 2.9 Data analysis

Data are presented as mean ± s.e.m. Statistical significance was tested using unpaired t-test to compare two separated groups of data. Experiments with more than two groups of data were analyzed by two-way ANOVA or one-way ANOVA followed by Dunnett's (for multiple comparisons against a single reference group) or Bonferroni's (for all pairwise comparisons) post hoc tests, as indicated in figure legends. Statistical analysis was performed using GraphPad Prism (GraphPad Software, San Diego, CA, USA).

## 3 Results

### 3.1 Pathways for NTBI entry in hippocampal astrocytes

**Fig. 1:**
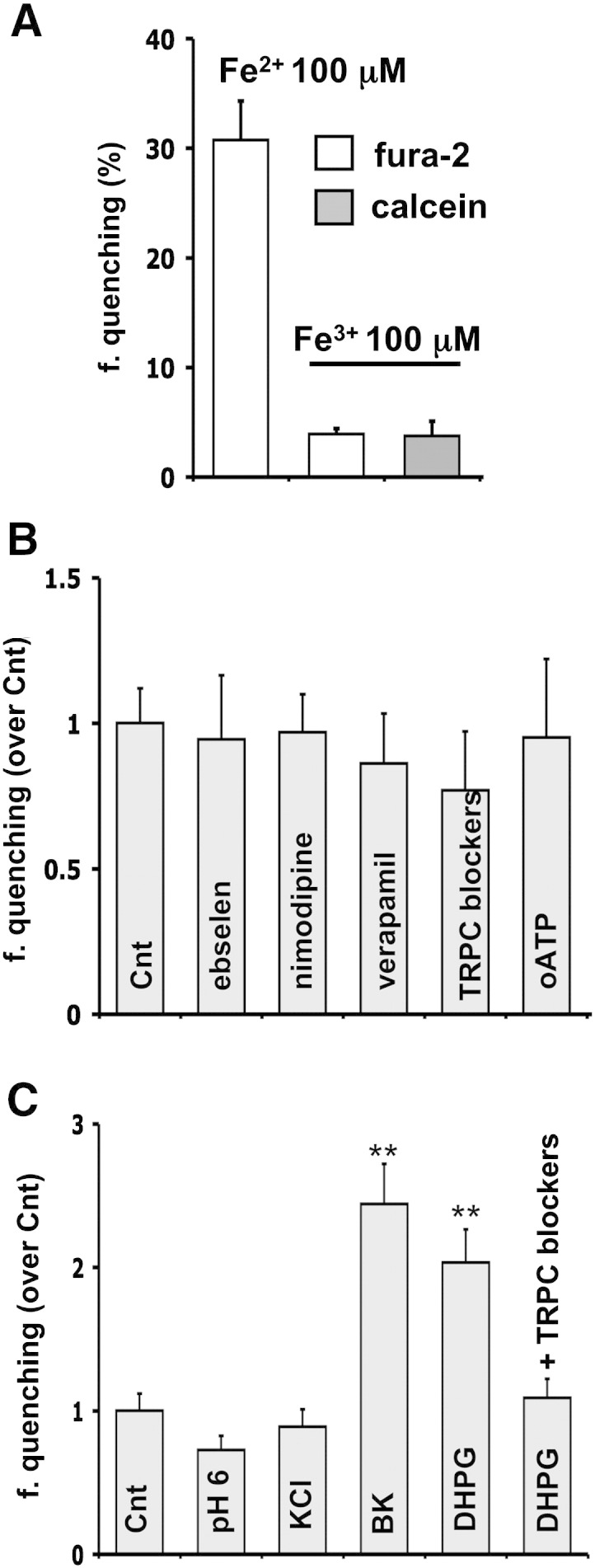
NTBI entry pathways in hippocampal astrocytes. Single cell imaging was performed on hippocampal astrocytes loaded with 4 μM fura-2 (355 nm excitation) and, when indicated, with 0.25 μM calcein (488 nm excitation). The iron-mediated quenching of the fluorescence signals, allowed the estimation of iron entry in different experimental conditions. The bars represent the mean values (± SEM) of fura-2 fluorescence quenching (f. quenching; ~ 20 astrocytes per experiment, 5–10 separate experiments per each condition). A: Fe^2 +^ uptake measurements. The sole administration of 100 μM Fe^2 +^ promoted a remarkable fura-2 quenching (with respect to basal values), while 100 μM Fe^3 +^ caused a much lower quenching of both fura-2 and calcein. B: Fe^2 +^ entry pathways. Fe^2 +^ entry in astrocytes was evaluated after administration of 5 μM Fe^2 +^, in control conditions as well as in the presence of the following blockers: 50 μM ebselen for DMT1; 10 μM nimodipine or 100 μM verapamil for VOOCs; 100 μM SC38249 together with 10 μM LU52396 for TRPCs; and 100 μM oATP for P2X7 receptors. None of these treatments significantly modified the fura-2 quenching observed in control condition. C: modulation of Fe^2 +^ entry pathways. Fura-2 quenching induced by 5 μM Fe^2 +^ was evaluated in control conditions as well as in the presence of different stimuli: pH reduction to 6 for DMT1; 30 mM KCl for VOCCs; and activation of metabotropic pathways, with either 100 nM bradykinin, BK, or 50 μM DHPG, for TRPCs. Only the indirect activation of TRPCs promoted a significant increase in Fe^2 +^ influx that was prevented by the mix of TRPC blockers (SC38249 and LU52396). Statistical significance in B and C was tested by one-way ANOVA followed by Bonferroni's post hoc test.

The first aim of this work was to investigate in pure hippocampal astrocytes the pathways potentially involved in NTBI entry. To this purpose, we performed a single cell fluorescence microscopy analysis, by exploiting the capability of iron to quench different fluorescent dyes [26], [27]. Among them, the calcium indicator fura-2 has recently drawn interest because of its selectivity for Fe^2 +^ when excited at the calcium-insensitive wavelength of 355 nm [15], [27]. The administration of 100 μM Fe^2 +^ (as ferrous ammonium sulfate; FAS) to fura-2 loaded astrocytes promoted a fast and marked fluorescence quenching (~ 30% reduction, 15 min after iron addition), comparable to neuronal counterpart [15], thus indicating the capability of astrocytes to uptake Fe^2 +^. Since the NTBI is expected to contain also ferric iron and considering that two recent studies have proposed an unidentified path for Fe^3 +^ entry in astrocytes [16], [17], we loaded these glial cells with both fura-2, which probes only Fe^2 +^, and calcein, a fluorescent dye sensitive to both Fe^2 +^ and Fe^3 +^ [26]. After incubation with 100 μM ferric ammonium citrate (FAC), fura-2 quenching was ~ 10 times lower than after administration of the same concentration of Fe^2 +^, thereby suggesting that a small fraction of Fe^3 +^ was reduced to Fe^2 +^ [17]. On the other hand, the evidence that the quenching of calcein was comparable to that of fura-2 further indicates that Fe^3 +^ ingress was negligible (Fig. 1A).

Based on this premise, we next used fura-2 microscopy to characterize the mechanisms responsible for the influx of Fe^2 +^ in astrocytes, by administering Fe^2 +^ in the presence of pharmacological treatments able to interfere with putative routes for iron uptake. In particular, in the following experiments, astrocytes were exposed to a more physiological concentration of Fe^2 +^ (5 μM), a condition that was able to promote a low, but reproducible, quenching of fura-2 (5.49% ± 1.53; ~ 30 cells per experiment from 10 separate experiments). Although the most credited mechanism of NTBI uptake in astrocytes is based on the iron transport through the DMT1, pre-incubation (45 min) with 50 μM ebselen, a selective blocker of the transporter [28] did not affect the process (Fig. 1B). Since in a previous work we demonstrated that calcium-permeable channels are the main responsible for iron entry in hippocampal neurons [15], we investigated whether this was true also for astrocytes. We first considered the contribution of voltage-operated calcium channels (VOCCs), which are reported to be expressed in astrocytes [29]. We evaluated the effects of two chemically distinct blockers of l-type VOCC, nimodipine (10 μM) and verapamil (100 μM). None of the two treatments significantly affected NTBI entry, even though verapamil reduced fura-2 quenching to some extent. We then considered the TRP canonical (TRPC) channels, which are permeable to calcium and are also expressed in astrocytes [30]. Cultures were treated with a mix of two blockers of TRPC: SC 38249 [31] and LU52396 [32]. Even in this case, a moderate reduction of fura-2 quenching was observed although not significantly different from controls. Finally, the amount of Fe^2 +^ entry was unaffected also by the oxidized ATP (oATP, 100 μM), a blocker of the P2X7 purinergic receptors [33] (Fig. 1B).

Overall, a major route for Fe^2 +^ influx did not emerge clearly in resting astrocytes, even though the TRPC channels appeared to exert some effect. In order to further explore this issue, we next assessed the relevance of these routes upon stimulation (Fig. 1C). In order to positively modulate the activity of DMT1 (a proton-coupled metal ion transporters), the pH of the extracellular solution was switched from 7.4 to 6; however, this condition did not improve the iron import, which, rather, was even slightly reduced. The administration of KCl (30 mM), to activate the VOCCs, promoted neither an influx of Ca^2 +^ (data not shown), nor an increase in Fe^2 +^ uptake. In contrast, the stimulation of the TRPC Ca^2 +^ channels, which are mainly activated by PLC-dependent depletion of intracellular calcium stores and by the increase in diacylglycerol, promoted a significant raise in Fe^2 +^ ingress. Indeed, PLC stimulation by either bradykinin (100 nM), a vaso-active peptide acting on B_2_ receptor or DHPG (50 μM), a selective agonist of group I metabotropic glutamate receptors, promoted an elevation of [Ca^2 +^]_i_ (not shown) and a significant increase in fura-2 quenching. This effect was specifically attributable to the opening of TRPC channels, since administration of the two blockers previously used (SC 38249 and LU52396) completely prevented the potentiation of Fe^2 +^ entry.

### 3.2 Effects of astrocyte activation on iron uptake

**Fig. 2:**
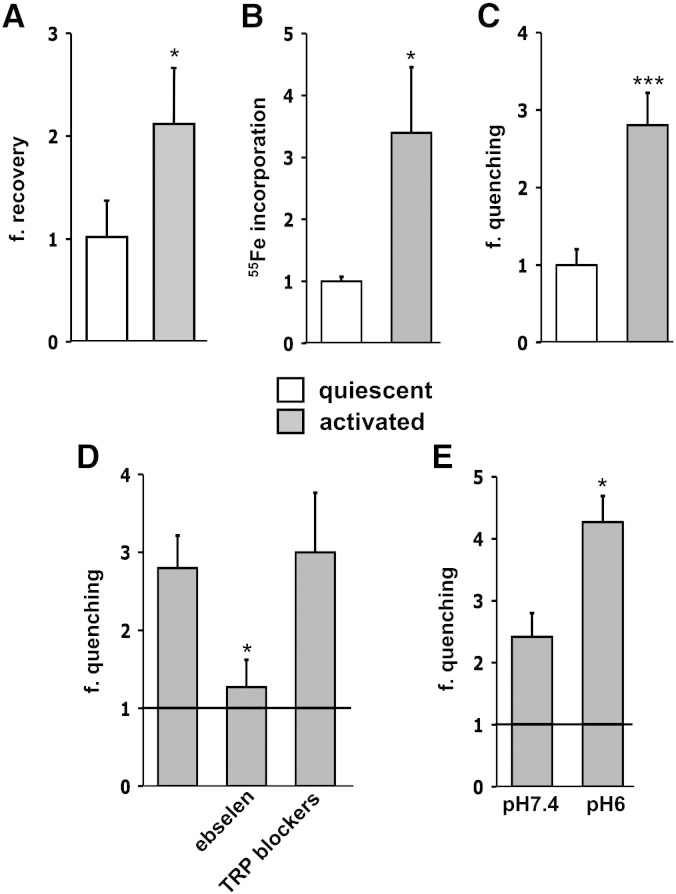
Effects of astrocyte activation on iron uptake. Astrocytes were activated by 24-hour treatment with 10 ng/ml IL1β and 30 ng/ml TNFα. In all panels, each bar was obtained by normalizing the values (expressed as mean values ± SEM) of activated over quiescent astrocytes. A: LIP measurements. LIP was estimated in terms of fluorescence recovery upon administration of the iron chelator (SIH, 100 μM) to calcein-loaded astrocytes. Fluorescence was measured by high throughput microscopy (HTM) in 4 separate experiments (~ 1000 astrocytes for each condition, per experiment). B: ^55^Fe uptake measurements. The uptake of ^55^Fe was quantified in lysates from astrocytes incubated for 30 min with 2 μM ^55^Fe. The counts per minute (cpm), corrected for protein content, were averaged from replicate samples in 2 separate experiments. C: Fe^2 +^ uptake measurements. The administration of 5 μM Fe^2 +^ promoted a quenching of fura-2 fluorescence significantly higher in activated compared to quiescent astrocytes (calculated in 10 separate experiments for both conditions). D: pharmacological modulation of Fe^2 +^ uptake in activated astrocytes. The DMT1 blocker ebselen (50 μM), but not the TRPC blockers (100 μM SC38249 together with 10 μM LU52396) prevented the potentiation of Fe^2 +^ entry induced by the activation process. The black line represents the reference quenching, after 5 μM Fe^2 +^ administration, in untreated quiescent cells. E: role of pH on Fe^2 +^ uptake in activated astrocytes. The acid pH further increased the Fe^2 +^ uptake in activated astrocytes. The values of fura-2 quenching were normalized as in D. Statistical significance was tested by: two-tailed paired t-test in A; two-tailed unpaired t-test in B, C and E; one-way ANOVA followed by Dunnet's post hoc test in D.

Inflammatory processes and neurotoxic conditions promote a microglia-dependent activation of astrocytes, with consequent changes in their phenotype and alterations in their physiological functions [34], [35]. Therefore, we investigated a possible effect of astrocyte activation on the mechanisms of iron uptake. In vitro activation was achieved by treating astrocytic cultures with 10 ng/ml IL1β and 30 ng/ml TNFα for 24 h and was assessed by quantifying specific activation markers [23] (IL6, nitric oxide, inducible nitric oxide synthase; data not shown). Upon activation, astrocytes showed an increase in the labile iron pool (LIP; Fig. 2A), which is an indication of an elevation of cellular iron level [36]. Accordingly, we evaluated the incorporation of iron in resting and activated astrocytes after 30 min exposure to two different concentrations of ^55^Fe (2 and 20 μM in the presence of 1 and 10 μM ascorbic acid, respectively). In the presence of 2 μM ^55^Fe (instead of 5 μM Fe^2 +^, because of the higher sensitivity of this assay compared to the analysis based on fura-2 quenching), the radioactive iron incorporation was more than doubled in activated astrocytes compared to those at rest (Fig. 2B), while at higher iron concentration there were no significant differences (not shown). These results were confirmed by the Fe^2 +^ uptake assay since, in activated astrocytes exposed to 5 μM Fe^2 +^, fura-2 quenching was two to three times higher than in controls, providing direct evidence of an increased iron ingress (Fig. 2C). In order to identify which iron influx pathway was potentiated upon activation, we evaluated the effects of the blockers above described. Ebselen, which previously had failed to affect basal iron entry, virtually abolished the potentiation of fura-2 quenching observed in cytokine-treated astrocytes, thereby suggesting that DMT1 is the main responsible for the increased Fe^2 +^ uptake in reactive astrocytes (Fig. 2D). In line with this hypothesis, the block of calcium permeable channels with nimodipine and oATP did not influence the effect of cytokine treatment (data not shown); interestingly, also the inhibition of TRPC channels, did not significantly reduce the fura-2 quenching in activated glial cells (Fig. 2D). As expected from these findings, also Ca^2 +^ influx was not increased upon astrocyte activation (not shown). Considering the putative role of DMT1 in potentiating iron uptake in activated astrocytes, we expected that a reduction of pH (from 7.4 to 6) could favor the activity of the uniporter, further amplifying its effect. Indeed, in the acidic environment a significant increase in fura-2 quenching was observed, thus indicating higher iron uptake (Fig. 2E).

### 3.3 DMT1 expression in inflammation-activated astrocytes

**Fig. 3:**
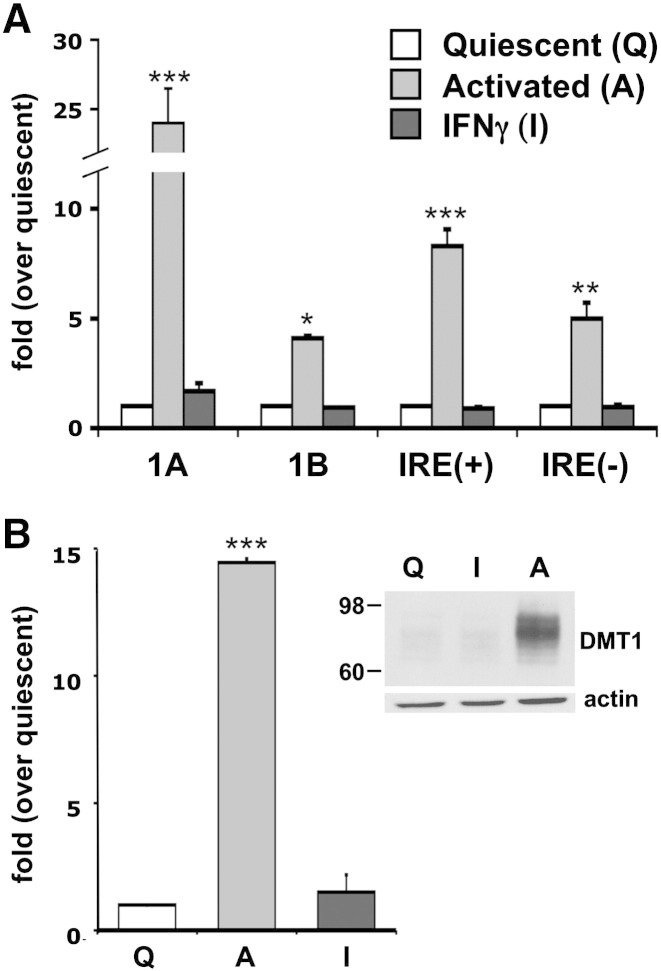
DMT1 expression in activated astrocytes. A: modulation of DMT1 transcripts by cytokines treatments. RT-qPCR analysis of DMT1 transcripts shows that the expression of all DMT1 isoforms is increased in astrocytes exposed to the protocol of activation (10 ng/ml IL1β and 30 ng/ml TNFα; 4 preparations), but not to 20 nM IFNγ (3 preparations). The DMT1-1A isoform appears to be the most affected by the activation process. B: modulation of DMT1 protein expression by cytokines treatments. The variations of the transcripts levels observed in A were paralleled by similar changes in the protein expression (revealed by an antibody that recognizes all DMT1 isoforms and normalized by actin). Inset: one of the 4 western blots analyzed in B. Statistical significance was tested by two-way (in A) and one-way (in B) ANOVA followed by Bonferroni's post hoc test.

Since DMT1 appears to be the entry pathway primarily responsible for the increased iron uptake observed in activated astrocytes, we next evaluated whether the cytokine-mediated activation was able to affect DMT1 expression, both at the transcript and at the protein level. Accordingly, we extended the RT-qPCR analysis performed on resting hippocampal astrocytes [25], to those exposed to the in vitro activation protocol. The primers, designed to discriminate the four different DMT1 isoforms at the C- and N-terminus, revealed an increase in the levels of all transcripts upon activation. Comparatively, there was a predominance of the DMT1-IRE(+) isoforms with respect to the IRE(−) and a significantly higher expression of the DMT1-1A isoforms – i.e. those responsible for Fe^2 +^ uptake at the apical side of duodenal enterocytes – with respect to the 1B (Fig. 3A). Of note, the treatment with interferon γ (IFNγ, 20 nM), another cytokine described to be effective in promoting an increase in DMT1 mRNA, at least in macrophages [37] and bronchial epithelial cells [38], failed to induce comparable effects on hippocampal astrocytes (Fig. 3A). The increase in DMT1 transcript levels observed in activated astrocytes, was accompanied by massive upregulation of the protein expression that, as expected, was not affected by the treatment with IFNγ (Fig. 3B).

**Fig. 4:**
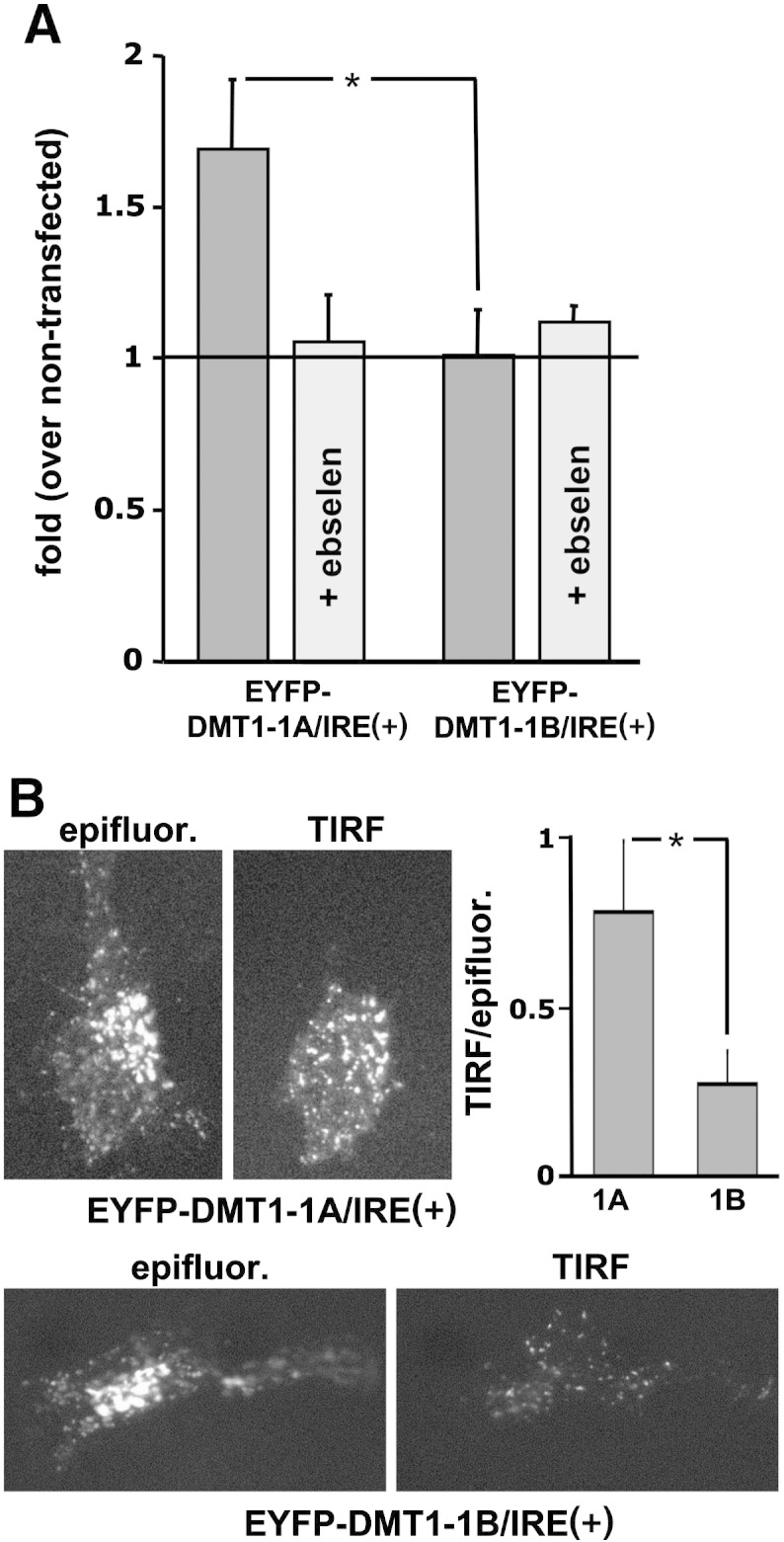
Overexpression of DMT1-1A and 1B in astrocytes. A: effects of DMT1-1A and 1B overexpression on Fe^2 +^ uptake. The overexpression of the EYFP-DMT1-1A/IRE (+) promoted an increase in Fe^2 +^ entry, induced by administration of 5 μM Fe^2 +^, with respect to non-transfected astrocytes (black line; 10 experiments). Pre-treatments with the DMT1 blocker (50 μM ebselen for 40 min) prevented the potentiation of Fe^2 +^ entry in transfected astrocytes (5 experiments). Over-expression of the EYFP-DMT1-1B/IRE (+) did not modulate Fe^2 +^ uptake and was not affected by ebselen treatment (7 experiments per condition). B: plasma membrane localization of DMT1-1A and 1B. Astrocytes transfected with EYFP-DMT1-1A/IRE (+) or EYFP-DMT1-1B/IRE (+) were analyzed by both epifluorescence and TIRF microscopy. The expression at the plasma membrane level was higher for EYFP-DMT1-1A/IRE (+) than for EYFP-DMT1-1B/IRE (+) as shown by the two pairs of images and by analysis of the ratio between TIRF and epifluorescence signals. Statistical significance was tested by one-way ANOVA, followed by Bonferroni's post hoc test, in A and by two-tailed unpaired t-test in B.

The relevance of DMT1-1A upregulation in sustaining iron uptake in activated astrocytes, was also supported by the results we obtained with astrocytes transfected with EYFP-DMT1-1A/IRE(+) and EYFP-DMT1-1B/IRE(+) respectively. While upregulation of EYFP-DMT1-1B/IRE(+) failed to modulate fura-2 quenching, the overexpression of EYFP-DMT1-1A/IRE(+) promoted an increase in Fe^2 +^uptake with respect to control cells analyzed within the same field of observation (Fig. 4A). As expected, the treatment with 50 μM ebselen was ineffective in the former condition, but completely prevented the positive effect played by DMT1-1A/IRE(+) on iron entry (Fig. 4A). Since the capability of DMT1-1A to mediate Fe^2 +^ influx in reactive astrocytes was expected to be due to its expression on the plasma membrane, we verified its localization with Total Internal Reflection Fluorescence (TIRF) microscopy. In fact, by this approach, the excitation of fluorophores occurs only within a narrow layer (~ 80 nm) juxtaposed to the coverslip, thus allowing to evaluate the fraction of the EYFP-DMT1 molecules located at the plasma membrane level. While conventional epifluorescence shows a similar signal pattern for both constructs, the TIRF images display a clear and punctate plasmalemma localization only for EYFP-DMT1-1A/IRE(+). The differences of the fluorescence levels showed by pairs of images acquired by the two methods were also quantified in terms of ratio analysis (Fig. 4B).

### 3.4 Effects of astrocyte activation on TfR pathway

**Fig. 5:**
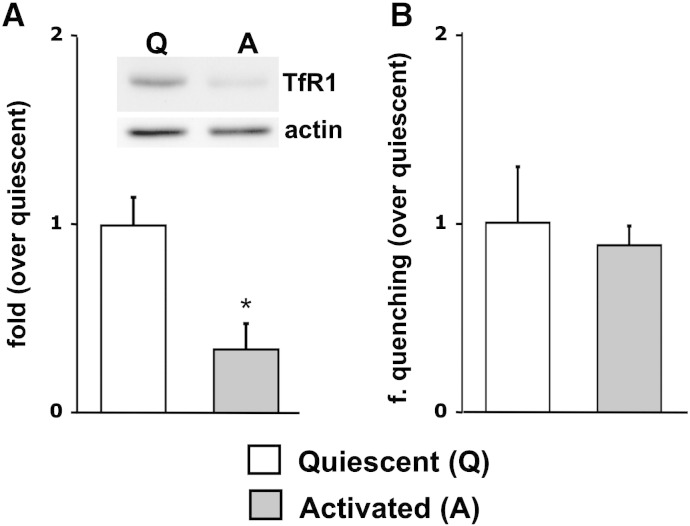
TfR1 in activated astrocytes. A: modulation of TfR1 expression. The activation process promoted a reduction of TfR1 expression (normalized by actin). Inset: one of the 4 western blots analyzed in A. B: modulation of TBI uptake. The activation did not affect fura-2 quenching after administration of 100 μg/ml TBI (B). Statistical significance was tested by two-tailed unpaired t-test.

Although the “in vivo” expression of TfR1 in astrocytes is still debated, the transcript as well as the protein were found in cultured cells [6], [39]. Since astrocyte activation induced the upregulation of the NTBI entry, we verified whether it similarly affected transferrin iron uptake: either directly, as a raise of TfR1 expression or, indirectly, as a consequence of the increase of DMT1, which could make more efficient the export of iron from the endosomes to the cytosol. Surprisingly, the western blot analysis revealed a significant reduction of TfR1 expression in activated astrocytes compared with the resting ones (Fig. 5A). On the other hand, the Fe^2 +^ uptake, revealed by quantification of fura-2 quenching, did not show any difference, indicating a similar cytosolic release of Fe^2 +^ from the endosomes during the TfR1 cycle, after administration of 100 μg/ml TBI (Fig. 5B). Also the quenching of calcein, whose fluorescence is affected by both Fe^2 +^ and Fe^3 +^, did not change between resting and activated astrocytes (not shown).

## 4 Discussion

The mechanisms responsible for iron entry in the various cell types of central nervous system (CNS) are still controversial and poorly characterized, although they are crucial for cellular iron handling and iron-dependent toxicity. This is particularly true for astrocytes that, in virtue of their specific morphology, play a crucial role in the regulation of iron flow from peripheral blood to cerebrospinal fluid (CSF) but may as well control iron concentration in the synaptic environment [6], [40], [41].

TfR1, which is responsible for the best-characterized route for iron entry, is reported to be expressed in astrocytes, but only in culture, not in vivo, at least in physiological conditions [6], [39], [42]. Our results not only confirm the expression and the presence of TfR1 in cultured hippocampal astrocytes, but also indicate that TBI does not significantly contribute to the amount of iron that enters these glial cells. It follows that the main source of iron for astrocytes is represented by the NTBI. This is expected to be mainly composed of Fe^2 +^, because of the highly reducing potential of the CSF [43], which is largely ascribable to the ascorbate released by astrocytes [16], and of the presence of iron reductases, such as Dcytb, SDR2 and STEAP2 [17], [44], [45].

Nonetheless, a recent study has also hypothesized an unidentified route for direct Fe^3 +^ influx in astrocytes [16]. Our data suggest that iron entry is mainly due to Fe^2 +^, with only a negligible contribution of Fe^3 +^, thereby opening the issue of which mechanisms are involved. The presence of DMT1 in the end-feet of in vivo astrocytes [46], [47] was put in relation to the regulation of brain iron absorption and this gave the basis to the idea that DMT1 might be expressed at the plasma membrane where it would control direct iron entry into the cytosol [40]. In contrast, we have recently demonstrated that the expression of DMT1 is very low in pure hippocampal astrocytes, both at the transcript and protein level [25]; moreover, our present results clearly show that this route does not play a significant role in Fe^2 +^ ingress. This draws the attention to mechanisms other than DMT1 for the cellular uptake of NTBI [17]. In the last decade there has been growing evidence that Fe^2 +^ can flow through Ca^2 +^-permeant channels in different cell types [13], [14], [15]. This was expected to be the case also for astrocytes, since they are endowed with l-type VOCCs, as well as members of the TRPC family and purinergic P2X7 receptors [48]. However, in control astrocytes, none of these routes appeared to be significantly involved in Fe^2 +^ entry, with the possible exception of TRPC, which showed a moderate reduction in Fe^2 +^ import when specific blockers were applied. Further consideration deserves the possible role of astrocytes in the control of iron within the synaptic environment, when they are exposed to physiological or pharmacological stimulation. This could be of particular relevance in excitatory synapses since, in the presence of a higher NTBI, glutamate release would favor the postsynaptic uptake of Fe^2 +^ through NMDA receptors and VOCCs [15]. Under these conditions, the astrocytic processes, which wrap the synapses, might shield neurons from this harmful event, with active iron sequestration. Of note, this process of iron clearance could be favored by the same synaptic activity, since spill over of glutamate from the synaptic cleft or accumulation of K^+^ might promote the activation of calcium permeant channels in astrocytes. However, the activation of VOCCs by physiological elevation of extracellular K^+^ concentration, did not induce an increase in Fe^2 +^ influx, in contrast to what we had observed in neurons [15]. The concomitant lack of [Ca^2 +^]_i_ variations provides further confirmation that VOCCs are not activated under these conditions; most likely, higher concentration of K^+^ is required, as it occurs when extracellular ionic osmosis is greatly perturbed [49]. These findings are in line with the report that K^+^-dependent Ca^2 +^ elevation in astrocytes is mainly due to the metabotropic response to the glutamate released by the neuronal activity [50]. Interestingly, the stimulation of different metabotropic pathways, via activation of group I metabotropic receptors or bradykinin receptors, displayed similar efficacy in determining iron influx. Therefore, our data indicate that TRPC channels, and not DMT1, play a central role in controlling NTBI import in hippocampal astrocytes, and that synaptic activity can modulate this process.

A central issue is the role astrocyte activation has in brain iron handling. In fact, under neuroinflammation or neurodegenerative conditions a change in astrocyte competence to control brain iron homeostasis could be crucial to confer them either a neuroprotective or neurodetrimental role [51], [52]. Our results showed that astrocyte activation, by an in vitro protocol that recapitulates the effects of in vivo inflammation, significantly altered iron handling, by increasing basal level of LIP and Fe^2 +^ uptake. A consistent body of evidence makes clear that this change in the functional phenotype was related neither to the TfR1 route, nor to calcium permeable channels. Indeed, a significant reduction of TfR1 expression was observed in activated astrocytes, as a direct consequence of cytokines action [53] or translational response to iron elevation. Rather, change in iron homeostasis could be ascribed to DMT1 as: 1) DMT1 was upregulated, at the level of both transcripts and proteins; 2) DMT1 blocker, but not TRPC blockers, completely reverted the potentiation of iron uptake; 3) lower extracellular pH further increased the ingress of iron, as expected for the DMT1 transporter that is energized by the H^+^ electrochemical gradient. These results are in line with recent evidence that TNFα is able to promote astrocyte activation with increase in DMT1 expression [54] and iron accumulation [55]. Conversely, the ability of IFNγ to enhance the DMT1 levels both in macrophages and bronchial epithelial cells [37], [38], was not observed in our hippocampal astrocytes.

In line with the expectations, RT-qPCR showed that the activation protocol promoted primarily the expression of DMT1-1A, i.e. the isoform mainly involved in iron intake at the level of the apical membrane of enterocytes [56], [57]. In agreement with this result, overexpression of DMT1-1A in control astrocytes led to its localization at the plasma membrane level, as revealed by TIRF microscopy, and favored iron entry. The expression of DMT1-1A after activation draws the attention to the role played by this transporter under acidosis. It is well known that extracellular pH can decrease at values even lower than 6 in acute states of brain inflammation, ischemic stroke and neurotrauma [58]. The above pathological conditions are potentially associated with astrocyte activation and may see extensive biodegradation of extravasated hemoglobin with consequent increase in the free iron concentrations within brain interstitial fluid [18]. Under these conditions, the expression of DMT1-1A in activated astrocytes and a strongly favorable H^+^ gradient are expected to significantly contribute to iron clearance. Of note, this competence pertains solely to DMT1 since acid-sensing ion channels are reported not to be expressed in the plasma membrane of hippocampal astrocytes [58], [59] while the activation of TRP vanilloid 1 (TRPV1) nocisensors [60] with capsaicin did not modulate the capability of astrocytes to uptake iron (not shown).

### 4.1 Conclusions

Overall, it emerges that astrocytes not only control iron access at the BBB level, but also buffer local extracellular iron changes at the synaptic level. In physiological conditions, glutamate spill over during neuronal activity can promote the activation of glutamate metabotropic receptor in astrocytes, thereby controlling iron uptake through the TRPC channels. Accordingly, the higher is the synaptic activity, the more efficient is also iron clearance in the synaptic environment, a mechanism that protects the synapse from iron overload, particularly when NTBI is higher. Under neuroinflammation, this control is potentiated by the process of activation that promotes the expression of DMT1-1A, further increasing astrocyte competence to uptake iron, particularly when acidosis establishes. In conclusion, if it is widely recognized that astrocyte activity is important to contain spill over of glutamate, we can now envisage that astrocyte can prevent also “spill in” of iron into the synaptic cleft, thereby protecting neurons from a potentially harmful iron overload through NMDAR and VOCCS. This complex control is expected to be relevant not only in iron dysmetabolisms but also in many neurodegenerative conditions, such as Alzheimer's, where neuronal hyperactivity, astrocyte activation and NTBI increase are established [61], [62], or Parkinson disease where the neuronal competence to uptake iron is reported to be potentiated [62], [63].
